# Effects of Light Interruption on Sleep and Viability of *Drosophila melanogaster*

**DOI:** 10.1371/journal.pone.0105678

**Published:** 2014-08-22

**Authors:** Zhenxing Liu, Zhangwu Zhao

**Affiliations:** College of Agronomy and Biotechnology, China Agricultural University, Beijing, China; Imperial College London, United Kingdom

## Abstract

Light is a very important regulator of the daily sleep rhythm. Here, we investigate the influence of nocturnal light stimulation on *Drosophila* sleep. Results showed that total daytime sleep was reduced due to a decrease in daytime sleep episode duration caused by discontinuous light stimulation, but sleep was not strongly impacted at nighttime although the discontinuous light stimulation occurred during the scotophase. During a subsequent recovery period without light interruption, the sleep quality of nighttime sleep was improved and of daytime sleep reduced, indicating flies have a persistent response to nocturnal light stimulation. Further studies showed that the discontinuous light stimulation damped the daily rhythm of a circadian light-sensitive protein cryptochrome both at the mRNA and protein levels, which subsequently caused disappearance of circadian rhythm of the core oscillator *timeless* and decrease of TIMLESS protein at nighttime. These data indicate that the nocturnal light interruption plays an important role in sleep through core proteins CRYTOCHROME and TIMLESS, Moreover, interruption of sleep further impacted reproduction and viability.

## Introduction

Sleep is an innate and complex biological phenomenon that helps organisms to adapt to the daily light/dark cycle, and environmental cues such as light and temperature can entrain the clock-cells which affect rhythmic behavior [1], [2], [3]. In animals, it is under debate whether the endogenous clock is affected by natural, dim nocturnal light, and most studies in mammals do not address the possibility that the clock may be highly light-sensitive, although a recent study in *Drosophila* demonstrated that flies are sensitive to low intensity light [4].

Because of the crucial role of the circadian system in timing different physiological processes of animals and in synchronizing them with the daily environmental changes (especially the changes in light), it is likely that clock dysfunction may have quite important effects on health [5]. Genetic or environmental disruptions of clock function accelerate physiological aging, onset of late-life diseases, and mortality risk in mammals [6], [7], [8], [9]. Many people in modern society routinely experience short sleep. For example, the National Sleep Foundation reported in 2010 that up to 27% of Americans obtained <6 h of sleep per day, mostly due to later bedtimes [10]. An additional reason for the later bedtimes and bad sleep episodes could be an increase in exposure to ambient light in the evening. Sleep problems are particularly prevalent in some persons with shift work, whose sleep disturbances and fragmented sleep are universal [11] .

*Drosophila melanogaster* exhibits all the behavioral characteristics of mammalian sleep, such as the timing of sleep onset, depth of sleep, and average duration [12], [13]. Sleep has been measured under various conditions, such as food deprivation [14], shadow stimulus [15], hypergravity [16], microgravity [17], and drug exposure [18]. The clock genes such as *per*, *tim*, *clk* and *cry* have been shown to affect sleep patterns in *D. melanogaster* [14]. In particular, one recent study showed that the blue light photoreceptive flavin-binding protein Crytochrome (CRY) is required for acute arousal upon sensory stimulation [19].

In flies, CRY targets TIM for degradation after lights on and synchronizes the circadian oscillations with external day/night cycles [20]. In addition to acting as circadian photoreceptor that mediates light input into the clock, CRY appears to function as a central clockwork component in peripheral clocks. In hypomorphic *cry^b^* null mutants, rhythmic expression of *per* and *tim* mRNA and their proteins is abolished in the peripheral clock cells but not in the central clock neurons [21]. It was shown that light-dependent transcriptional and post-translational activity regulates most endogenous CRY in *Drosophila*.

Therefore, in this study, we explored the effects of discontinuous light stimulation (DLS) during the night on CRY, CRY-related core oscillator TIM, sleep, and several physiological functions in *D. melanogaster*, in order to understand the sleep and physiological disorders that are produced by a light signal that mimics the disruption produced by light in modern human societies.

## Materials and Methods

### Fly rearing and fly strains

Flies were maintained in a 12 hL/12 hD photoperiod, 60% relative humidity and 25°C on a modified cornmeal/agar medium. The genetic background of all strains was from the w^1118^ strain except for tim-Gal4 and Cry-Gal4 that have yw background. The UAS-tim-RNAi and UAS-cry-RNAi were obtained from Center of Biomedical Analysis, Tsinghua University. The null mutants cry^b^ (background Canton S) and tim^01^ (background w^1118^) were obtained from laboratory of Dr. Rosbash. Light intensity was 500–600 lux. By convention, the time of lights-on was denoted as ZT0 (Zeitgeber time) and the time of lights-off as ZT12. In accordance with the natural environment, lights were turned on at 06:30 h (ZT0) and off at 18:30 h (ZT12).

### Behavioral analysis of sleep

Sleep was continuously monitored and recorded in 1 min bins by placing individual adult flies (3 to 12 day old males) in glass tubes and monitoring their activity using the Drosophila Activity Monitoring System (DAMS) and Data Acquisition System (DAS)(Trikinetics, Waltham, MA). Black boxes were fitted with light-emitting diodes. The light intensity inside the boxes was adjusted using an Atten (Shenzhen, China) regulated DC power supply and detected with a Trikinetics environmental monitor. Before being placed in the locomotor monitor, flies were entrained in LD cycles in the same incubator, with lights-on at 06:30 h (ZT0). Subsequently, starting on day 3, treat flies were exposed to DLS (white light, 500 lux, and 0.008 mW/cm^2^ for 1-h intervals) at different time points (four light stimulations: ZT13–14, ZT 15.5–16.5, ZT18–19 and ZT20.5–21.5) for 4 days, and their sleep was recorded (Fig S1). We choose this condition to make sure that CRY was fully degraded due to DLS .Controls were flies without any DLS light interruption.

Generally, flies subjected to LD 12∶12 cycles were given a day for adaptation, and data from 4 days of DLS treatments and 4 days of subsequent recovery/rebound periods were used for analysis. The average total sleep, the average sleep episode duration and the sleep bout number were calculated based on the sleep definition as a period of 5 or more minutes of behavioral immobility [22], [23].

### Quantitative Real Time PCR

Total RNAs from 20 male brains of the treatment and control groups were collected at the same circadian times. Total RNA was extracted using Trizol, treated with DNase I and reverse transcribed to cDNA using a Quantscript RT kit (Tiangen Biotec Co., Ltd., Beijing, China). The quantitative PCR assay was performed using Applied Biosystem 7500 and 7300 Real Time PCR systems (Applied Biosystem, Foster, CA, USA). The PCR mixture contained Real master Mix (SYBR Green I) (Tiangen Biotec Co., Ltd., Beijing, China), double-distilled H_2_O and the following primers: actin primers, 5′-CAGAGCAAGCGTGGTATCCT-3′ and 5′-CTCATTGTAGAAGGTGTGGTGC-3′; *cry* primers, 5′- AGGATTTGCATAGAGCAGGACT -3′ and 5′- GCAGGAACATTTGGTAGGTCA -3′; *tim* primers, 5′-TTCTCCTCCTTGGGTT GCTT-3′ and 5′- ATTCTCCAGCAGCGGTATCA-3′. Cycling parameters used for *cry* were 95°C for 3 min, followed by 40 cycles of 95°C for 15 s and 60°C for 1 min. The target gene mRNA level was normalized against the actin level. The 2-Ct (comparative Ct method) stand curve method was used [24]. The mean of three to four independent replicates was determined.

### Immunoblot Analysis

Flies were synchronized for 3 days under LD (500 lux:0 lux) cycles, and collected every 3–4 h. Protein was extracted from 40–50 male heads at each time point. Frozen heads were homogenized in 150 ml ice-cold extraction buffer (20 mM HEPES, pH 7.5, 100 mM KCl, 5% glycerol, 10 mM EDTA, 0.1% Triton, 1 mM dithiothreitol, 0.5 mM phenylmethylsulfonyl fluoride) plus protease inhibitors (complete mini Roche), phosphatase inhibitors cocktails 1 (0.5%) and 2-mM b-glycerophosphate (1%; Sigma). For SDS–PAGE, 50 ug of protein extracts were separated on 8–10% Tris–acetate gels (Invitrogen) and transferred to NC membranes. Primary antibodies used were mouse anti-ACTIN (abcam;1∶2000) and rat ani-CRY and rat anti-TIM (from the laboratory of Dr. Rosbash Lab; anti-CRY 1∶500; anti-TIM 1∶1000). HRP-conjugated secondary antibodies (Zhongshan Goldenbridge Biotechnology Co., Ltd) were goat anti-mouse IgG (1∶1000) and goat anti rat IgG (1∶1000). All western blots were performed in triplicate. Band intensity was calculated and analyzed with the Gel-Pro Analyzer 4.0.

### Assays of egg-laying amount in female flies treated with DLS

In order to count the numbers of eggs, freshly eclosed flies were collected and reared at concretionary apple Juice-Agar Medium covered by a thin layer of active dry yeast paste. All these individuals were normally reared under LD condition, except for the flies in treatment group which were treated with DLS during every night. The apple Juice-Agar Medium was prepared by boiling 5 L of water containing 200 g agar, cooled down to about 50–55°C, then adding 1.6 L apple juice, 165 g dextrose, and 9 g Tegosept which had been dissolved in 50 ml 70% ethanol. The mixture was stirred for 15 minutes, and 300 ml of apple Juice-Agar Medium were poured into the trays. The medium was allowed to cool to room temperature. The trays were enclosed in a plastic bag and stored at 4°C until used. Before use, the Apple Juice Agar Medium in the trays was added with active dry yeast paste. Female flies lay eggs on the food in the trays while eating. The food trays were removed and replaced with new trays every 24 h for counting eggs under microscope. Total 96 female flies (half treatment vs half control) were used for egg-laying study, in which 24 females/each repeat and four replicates were carried out.

### Viability assay of flies treated with DLS

This experiment was explored with 800 freshly eclosed fly individuals (half treatment vs half control) at triplicates. All these individuals were normally reared under LD condition, except for the flies in treatment group which were treated with DLS during every night. The flies were maintained on SCM (standard cornmeal-yeast-agar medium) at 25°C and 60% humidity, and refresh the medium every day. Numbers of survival and death of fruit flies were recorded every 24 h for survival rate, and removed dead flies.

### Statistical analyses

All statistical analyses were performed by using SPSS software (version 11.5); one-way ANOVA was used, followed by a LSD analysis; p<0.05 was regarded as significant.

## Results

### Ambient light affects sleep in wildtype w^1118^

**Figure 1:**
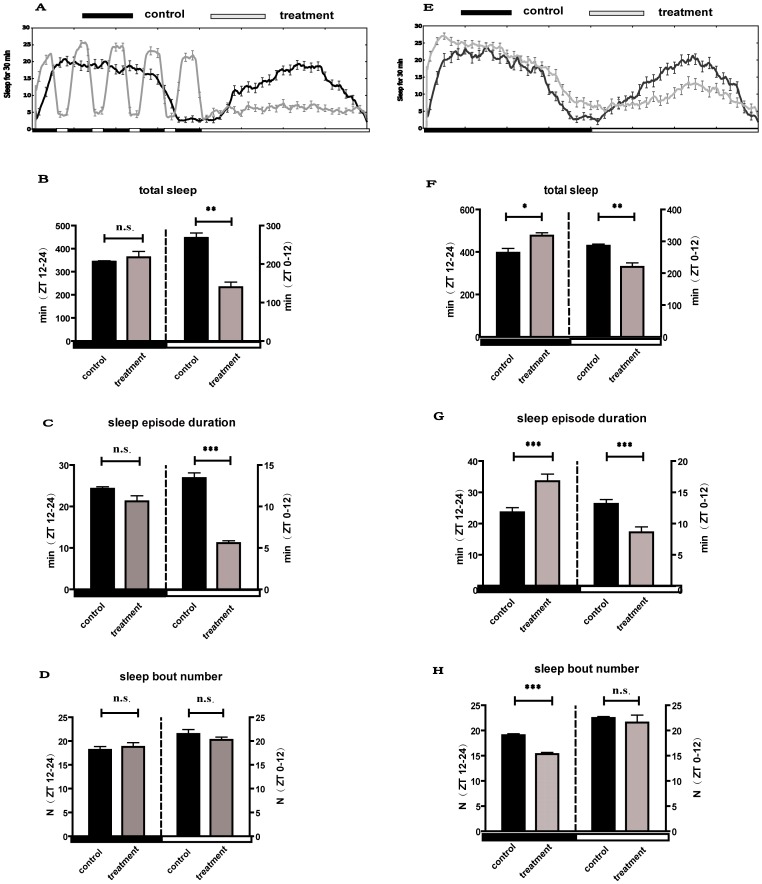
Sleep profiles of the control and light treated flies during DSL and during recovery. A–D indicate sleep profiles during DLS. (A) Sleep profiles in treated (DLS) and control (normal LD condition) flies (n = 96 for all groups). (B) Total sleep during the day (ZT 0–12) and night (ZT12–24). (C) The average sleep episode duration during the day and night. (D) Average sleep bout numbers. E–H indicate sleep profiles during the recovery period after DLS. (E) Sleep profiles in treated and control (normal LD condition) flies (n = 96 for all groups). (F) Total sleep during the day and night. (G) The average sleep episode duration during the day and night. (H) Average sleep bout numbers. White and black boxes under the x axis indicate light and dark periods of LD, respectively. *: p<0.05; **: p<0.01; ***: p<0.001, and the bar heights indicate mean values ± s.e.m.

To address the role of light disruptions in *Drosophila* sleep, we examined the effect of DLS on sleep. During light interference of 4 days, the average total sleep per 24 h in males was only marginally reduced (table 1, p = 0.056), in which sleep was strongly reduced during the daytime (p<0.01) but not the nighttime (p>0.05), even though the nighttime sleep rhythm was disrupted by the wake periods induced acutely in response to DLS (Fig 1A–B). The light-treated males displayed a 48% decrease in total daytime sleep, caused by a decrease in sleep episode duration (p<0.001) but not sleep bout number (p>0.05) (Fig 1C–D). These results indicate that DLS strongly disrupt *Drosophila* sleep, especially impacting decreases of daytime sleep.

**Table 1:** Effects of DLS on sleep.

Sleep assay period(days)	Group	Daily total sleep (min)	p value	Sleep episode duration (min)	p value	Sleep bout number (N)	p value
During DLS(4–7)	control	616.54±22.36	P<0.05	37.69±0.50	P<0.01	39.59±1.62	P>0.05
	DLS treatment	501.08±57.21		26.82±1.33		38.99±1.02	
After DLS(8–11)	control	682.29±27.16	P>0.05	36.77±1.78	p>0.05	41.54±0.19	p<0.01
	DLS treatment	696.47±46.07		42.17±3.12		36.89±1.16	

Moreover, during the recovery period (12 h L:12 h D without DLS) of four days after the 4-day DLS period, although the total sleep per 24 h in treated male flies did not differ from that of untreated controls (table 1, p>0.05), the nighttime sleep was significantly enhanced in the DLS flies (p<0.05) (Fig 1E–F), with increases of sleep episode duration (p<0.001) (Fig 1G) and decreases of sleep bout number (p<0.001) (Fig 1H), indicating improved sleep quality at nighttime during the rebound period. In contrast, the treated flies (during the rebound period) slept less in the daytime than control flies, with a 23% decrease in sleep (p<0.01) (Fig 1E–F), and it was caused by reduced sleep episode duration (p<0.001) (Fig 1G) but not sleep bout number (p>0.05) (Fig 1H). These data suggest that the effect of DLS on sleep seems to be long-term and a cumulative effect, in which sleep during the scotophase increases and sleep of the photophase decreases during the recovery/rebound stage.

### The oscillations of cry gene products are dampened by discontinuous light stimulation

**Figure 2:**
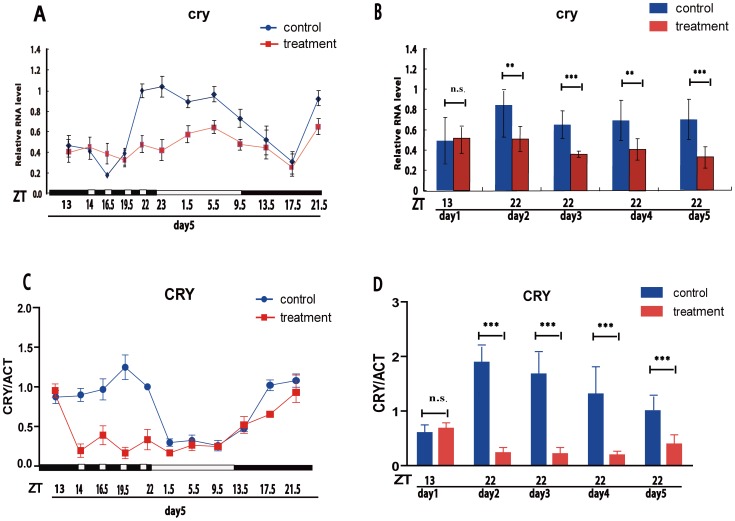
The oscillations of *cry* gene products are dampened by DLS. (A) Quantitative RT-PCR shows that cryptochome (*cry*) RNAs cycle robustly in abundance in the brains of control (black line) and less robustly in treated flies (red line). (B) *cry* mRNA levels at ZT22 (initiation of after light treatments) of different days in control (black columns) and treated flies (red columns), and at ZT13 of day 1 before treatment as a baseline. Immunoblots were carried out with an antibody for CRY or ACTIN at different times of days on the indicated day after initiation of treatment. (C) CRY protein amounts were normalized to ACTIN values for all time points. Each curve represents three independent measures of protein abundance from three independent fly head extracts. Error bars indicate standard error. (D) CRY protein expression level at ZT22 (after light treatments) of different days in control (black columns) and treated flies (red columns) in order to detect the expression conditions of different days, and ZT13 of day 1 before treatment as a baseline. ***: p<0.001.

We assayed daily mRNA expression profiles of *cry* in the adult brains of wild-type male flies in a 12L:12D cycle and a discontinuous light stimulation (DLS) treatment, respectively. Results showed that *cry* mRNA in wild-type flies in a normal LD cycle exhibits a daily oscillation, with a peak at Zeitgeber time (ZT) 4–8 and a trough at ZT 16, as previously observed [25]. By contrast, the daily oscillation was significantly dampened in the DLS flies (Fig 2A). To make sure that the *cry* mRNA did not change before light stimulation, we detected the *cry* mRNA level at ZT13 during the first day for both the control and DLS flies (prior to DLS treatment) as a control point, and found that they have the same transcript level (Fig. 2B). Moreover, we also detected the *cry* mRNA level in the three light-stimulated days at the same time point (ZT22) (after DLS treatment). The result show that the *cry* mRNA levels were significantly reduced in the treated flies on all three days (Fig. 2B).

Furthermore, we determined protein levels of CRY at different time points for several days by immunoblot analysis. Results showed that CRY also exhibited a daily oscillation during 24 h in wild-type heads from flies maintained in a normal LD cycle (Fig 2C–D), with the highest levels near the end of the dark phase (ZT19.5) and a trough during the daytime (ZT 9.5), as has previously been observed [25], [26]. While flies were treated with intervals of light (1 h/each time) during the night, the levels of CRY were strongly reduced across all time points during the night, suggesting that CRY was degraded by light (Fig 2C–D). As with the analysis of *cry* mRNA, we analyzed the expression level of CRY at ZT13 in both treated and control flies on the first day before light stimulation (as a basic point) and also analyzed the CRY protein level in the three days of DLS at the same time point (ZT22) for both groups. The results of the CRY protein analysis were is consistent with the analysis of *cry* mRNA levels (Fig 2C, 2E) and demonstrated that DLS produced a stably dampened oscillation of CRY protein.

### The oscillations of *tim* gene products are dampened by DLS

**Figure 3:**
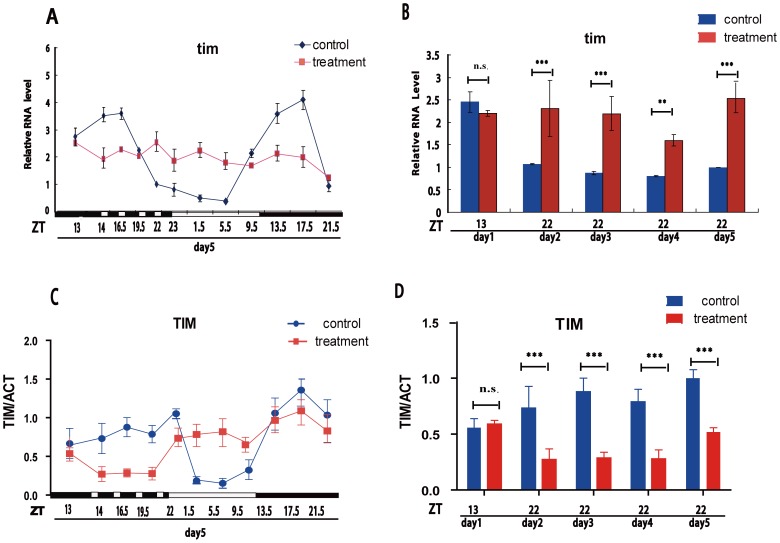
The oscillations of *tim* gene products are changed by DLS. (A) Quantitative RT-PCR shows that *timeless* (*tim*) RNAs cycle robustly in abundance in the brains of control (black line) and less robustly in treated flies (red line). (B) *tim* mRNA levels at ZT22 (initiation of after light treatments) of different days in control (black columns) and treated flies (red columns), and at ZT13 of day 1 before treatment as a baseline. Immunoblots were carried out with an antibody for TIM or ACTIN at different times of days on the indicated day after initiation of treatment. (C) TIM protein amounts were normalized to ACTIN values for all time points. Each curve represents three independent measures of protein abundance from three independent fly head extracts. Error bars indicate standard error. (D) TIM protein expression level at ZT22 (after light treatments) of different days in control (black columns) and treated flies (red columns) in order to detect the expression conditions of different days, and ZT13 of day 1 before treatment as a baseline. ***: p<0.001.

CRY is acutely sensitive to light. It would be crucial to determine if the oscillations of the core oscillator are impacted, we further analyzed *tim*/TIM in both RNA and protein levels like cry/CRY assays above. Results showed that *tim* mRNA in wild-type flies in a normal LD cycle exhibits a daily oscillation, with a peak at ZT 13–16 and a trough at ZT 5, as previously observed [25], [26], but the daily oscillation of *tim* was significantly dampened without any rhythm in the DLS flies (Fig 3A–B). Subsequently, the peak of TIM at night in control flies was greatly reduced in DLS flies (Fig 3C–E). These data indicate that the DLS changed this core clock gene oscillation.

### Sleep patterns in null mutant cry^b^ and tim^01^

**Figure 4:**
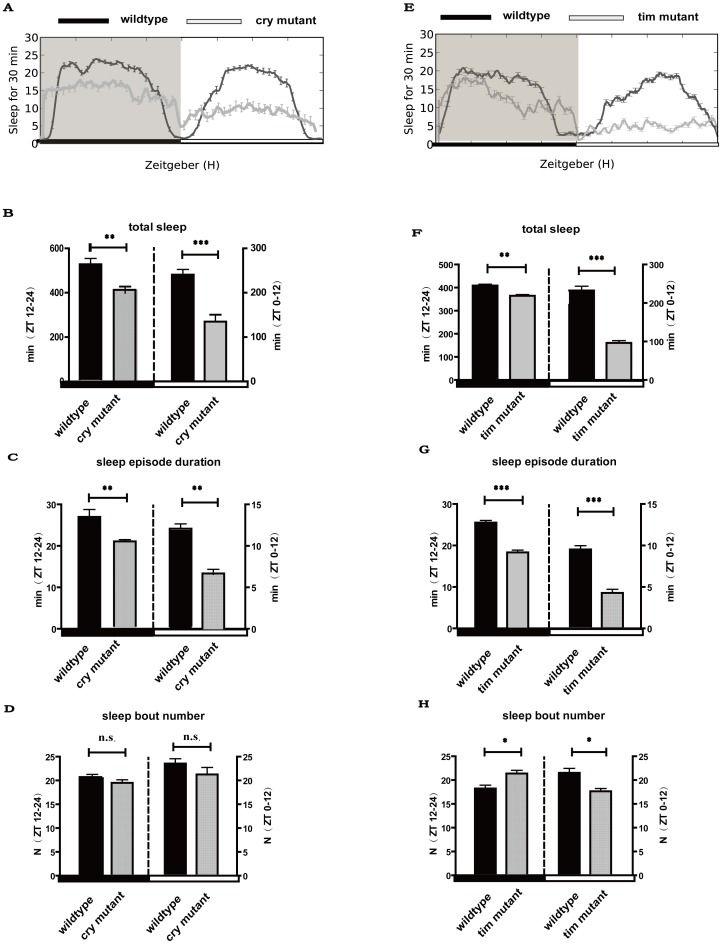
Sleep patterns in null mutant *cry^b^* and *tim^01^*. A–D indicate sleep profiles during LD condition in null mutant *cry^b^*. (A) Sleep profiles in null mutant cry*^b^* and wild type flies (n = 96 for all groups). (B) Total sleep during the day (ZT 0–12) and night (ZT12–24). (C) The average sleep episode durations during the day and night. (D) Average sleep bout numbers. E–H indicate sleep profiles during LD condition in null mutant *tim^01^*. (E) Sleep profiles in null mutant tim^01^ and wild type flies (n = 96 for all groups). (F) Total sleep during the day (ZT 0–12) and night (ZT12–24). (G) The average sleep episode durations during the day and night. (H) Average sleep bout numbers. White and black boxes under the x axis indicate light and dark periods of LD, respectively. *: p<0.05; **: p<0.01; ***: p<0.001, and the bar heights indicate mean values ± s.e.m.

Because the DLS changed daily oscillations of the core clock genes *cry* and *tim*, by suppressing both CRY and TIM expression levels at night. The phenotype caused by DLS in wild type should coincide with the *cry* and *tim* null mutant strain. To address this conjecture, we directly examined the sleep patterns in null mutants cry^b^ and tim^01^. Results showed that the total sleep at nighttime and daytime in both cry^b^ and tim^01^ mutants significantly decreased (p<0.01 and p<0.001 separately at nighttime and daytime) (Fig 4A–B & Fig 4E–F) mainly caused by sleep episode duration (Fig 4C–D & Fig 4G–H). The daytime behavioral phenotype of mutant*s* cry^b^ *and* tim^01^ is similar to the effects of DLSin wild type caused by decreases of CRY and TIM levels at night. These results indicate that the nocturnal light disrupt *Drosophila* sleep mainly through CRY and TIM proteins.

### Loss of functions of *cry* and *tim* affect sleep

**Figure 5:**
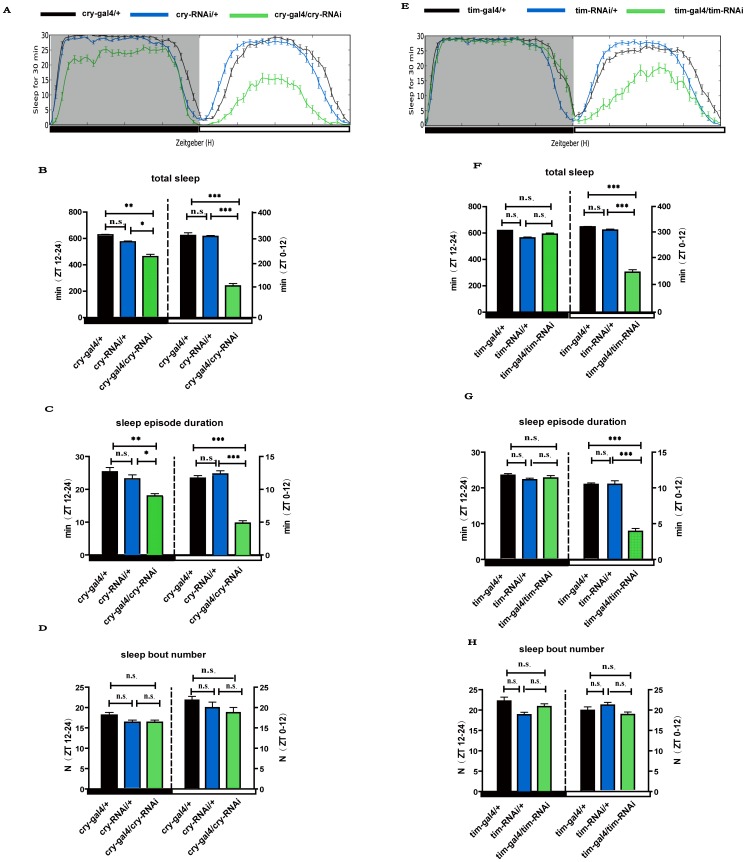
Loss of functions of *cry* and *tim* affect sleep. A–D indicate sleep profiles during LD condition in loss *cry* of function. (A) Sleep profiles in cry-RNAi and their control flies (n = 64 for all groups). (B) Total sleep during the day (ZT 0–12) and night (ZT12–24). (C) The average sleep episode durations during the day and night. (D) Average sleep bout numbers. E–H indicate sleep profiles during LD condition in loss *cry* of function. (E) Sleep profiles in tim-RNAi and their control flies (n = 64 for all groups). (F) Total sleep during the day (ZT 0–12) and night (ZT12–24). (G) The average sleep episode durations during the day and night. (H) Average sleep bout numbers. White and black boxes under the x axis indicate light and dark periods of LD, respectively. *: p<0.05; **: p<0.01; ***: p<0.001, and the bar heights indicate mean values ± s.e.m.

We used RNAi to explore the effects of loss of function of *cry* and *tim* on sleep regulation. Consistent with the short sleep phenotype in the mutant cry^b^ flies, total sleep in the cry > cry-RNAi flies was significantly less than the control flies (Fig 5A–B), and the sleep episode duration was lower than the control flies at both daytime (p<0.001) and nighttime (p<0.05) while the sleep bout number was not changed (P>0.05) (Fig 5C–D). Similarly, the tim > tim-RNAi loss-of-function flies slept less than the control flies mainly due to decreased total sleep during daytime (p<0.001) (but not at nighttime) (Fig 5E–F) mainly caused by the sleep episode duration (p<0.001) (Fig 5G), while sleep bout number was not changed (P>0.05) (Fig. 5H). These results further indicate that CRY and TIM are important to maintain normal sleep.

### Effects of light interruption on egg-laying

**Figure 6:**
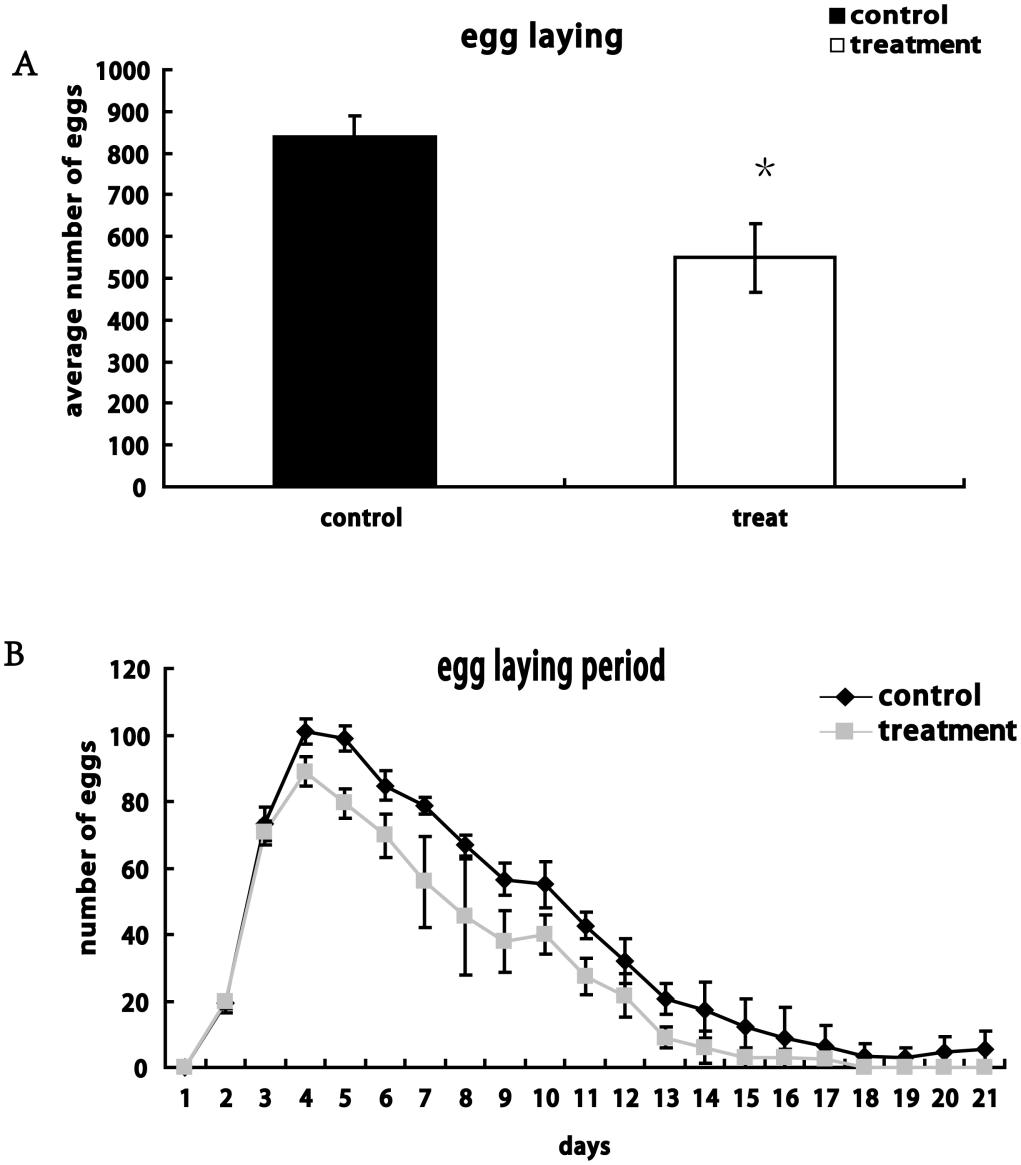
Effect of DLS on the egg-laying of female flies. Single virgin female flies were crossed with two males for analysis of egg-laying. (A) Compared to the control flies (black column), DLS-treated flies (white column) lay fewer eggs (p<0.05) during entire egg-laying period. (B) DLS depresses egg-laying of flies during the whole egg laying period. *: p<0.05.

To determine the effects of DLS on egg-laying in *Drosophila*, we examined differences in daily egg-laying quantity between control and treated females. Results indicated that the females subjected to DLS had a lower egg-laying quantity than controls, by decreasing 34.4% compared to the egg-laying of controls (Fig 6A) during whole egg-laying stage of females. Importantly the daily egg-laying quantity in DLS treatment flies was less than that in control flies (Fig 6B). These data demonstrate that DLS treatment results in lower fecundity in *Drosophila*.

### DLS affects viability

**Figure 7:**
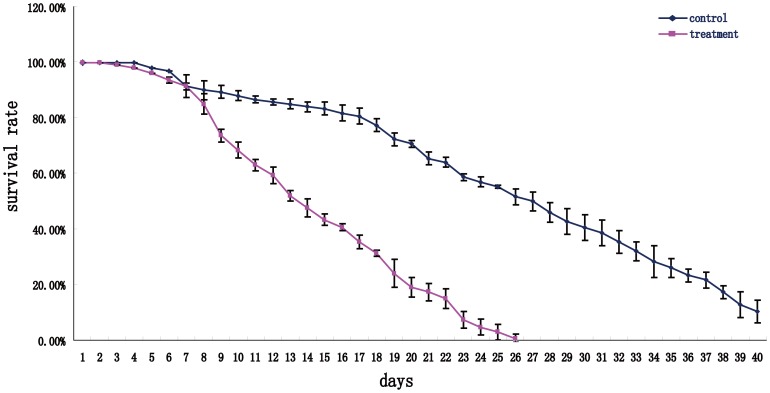
Effects of DLS on survival and viability. Survival rate of males at different days in control (blue line) and treatments (red line), which was calculated by below formula. Survival rate =  surviving fly number/initial fly number ×100%.

One recent study showed that overexpression of CRY helps slow down the aging process and reverse age-associated phenotypes [27]. To determine the relationship between DLS and viability, we compared the fly survival level over the whole lifespan. Results showed that DLS significantly caused decreases of survival rates (Fig 7), which indicates that DLS-induced disruptions to the circadian system reduce the viability of flies.

## Discussion

Circadian clocks regulate daily rhythms in behavior, physiology, and cellular processes, ensuring homeostasis coordinated with day/night cycles [28]. Daily physiological and behavioral oscillations have been extensively studied in a wide variety of organisms, ranging from bacteria to human. These circadian oscillations driven by circadian clocks keep running under constant environmental conditions, but can be reset by environmental stimuli such as light [29].

In flies, the blue light photoreceptive flavin-binding protein Crytochrome (CRY) is crucial for the synchronization of individual oscillator cells [30]. CRY is degraded after light interruption, resulting in a daily reset and adaptation of the circadian clock to its environment. CRY contains a flavin and a pterin-binding pocket, and its activation causes intra-molecular electron transfer [31], [32]. If the cellular redox system is compromised, light-induced CRY degradation is impaired. The previous study showed that cellular redox status and electron transfer modulate light-dependent activation of CRY, which in turn affects subsequent transmission of the light signal to TIM and the degradation of CRY itself [31]. In this study, we found that firstly light interruption at scotophase eliminated the circadian rhythm of CRY expression, which finally eliminated the circadian rhythm of TIM expression. In the end sleep analysis found that light interruption directly reduced daytime sleep by decreasing average sleep episode duration but not the sleep bout number, and subsequent recovery/rebound periods after light interruption improve the quality of nighttime sleep and decrease the quality of daytime sleep. These results indicate that nighttime light-driven elimination of the clock protein CRY and TIM cause changes in sleep as well as general organismal and reproductive fitness, as functions of TIM previously reported [33]. A crucial function of CRY and TIM would therefore be to maintain the normal sleep pattern of *Drosophila,* which is consistent with the previous report that CRY is required for acute arousal upon sensory stimulation [19]. Moreover, the DLS treated phenotype (caused by decreases of CRY and TIM at night) and tim-RNAi phenotype (both causing TIM reduction but not deficiency) have same sleep pattern by decreasing sleep only at daytime, but the null mutant tim^01^ phenotype decreases sleep at both daytime and nighttime. These results suggest that sleep amount at daytime is much sensitive to TIM level. Further, TIM is required for PER stability, and both of them have same expressive patterns.

Sleep disorders usually cause acceleration of aging, health problems and some other defects in physiology [34]. It has reported that *D. melanogaster* TIM is important for the establishment of species-specific behavioral rhythms such as the mating activity rhythm [33]. It was also reported in *Drosophila* that aging and reproduction are associated with sleep changes [35] and these sleep changes are associated with dampened molecular oscillations of core clock genes [36]. Longevity is influenced by various environmental conditions, including temperature, oxidative stress, food composition and population density/social interactions [37]. In addition, a voltage-gated potassium channel mutant (*shaker*) that decreases the amount of sleep has reduced longevity [38], [39]. In this study, we established a novel relationship between nighttime light interruption and sleep and longevity, and the results suggest that elimination of CRY rhythms caused by light interruption at scotophase results in degradation of TIM and elimination of TIM rhythms that causes decreases in total sleep, and the latter cause reduction of fecundity and shortening of longevity.

## Supporting Information

Figure S1 **The DLS treatment.** Adult flies were entrained in LD cycles in the same incubator, with lights-on at 06:30 h (ZT0) and lights-off at 18:30 (ZT12). Subsequently, during day 3, control flies were always in the same condition (12L:12D), but treated flies were exposed to discontinuous light stimulation (DLS) during scotophase. We used light interruption to treat flies (white light, 500 lux, and 0.008 mW/cm2) for one hour and intervals of one and half hours between treatments, with four repeats (treatment times: ZT13–14, ZT 15.5–16.5, ZT18–19 and ZT20.5–21.5) delivered daily for 4 days, during which their sleep was recorded. We choose this condition to make sure that CRY was fully degraded due to DLS but to maintain a rhythm in sleep.
(TIF)
