# Supplementary figures and images for: Effects of Light Interruption on Sleep and Viability of Drosophila melanogaster
Source: PLoS One. 2014 Aug 22;9(8):e105678. doi: 10.1371/journal.pone.0105678 (PMC4141813; doi:10.1371/journal.pone.0105678)

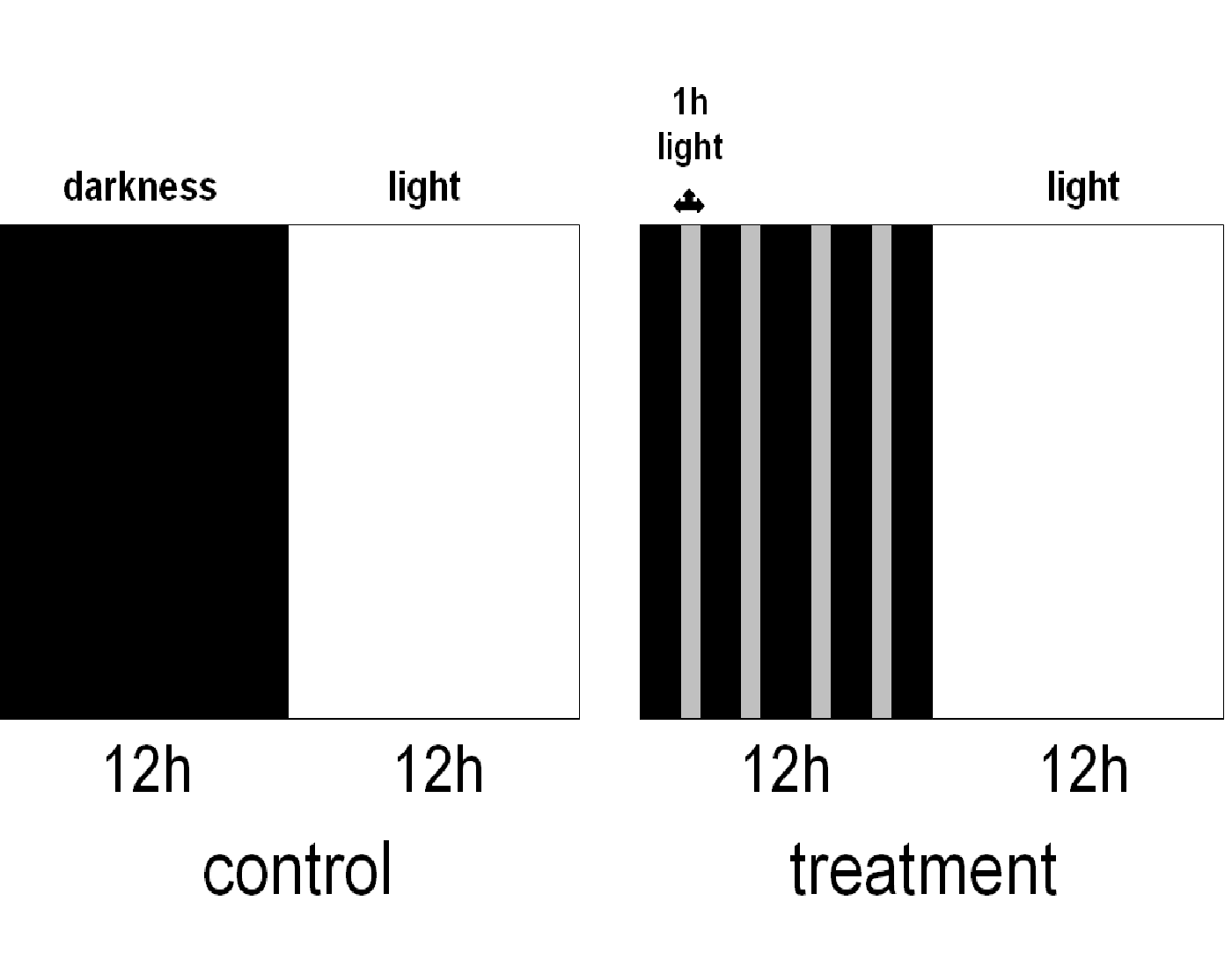

Supplement: Figure S1 — The DLS treatment. Adult flies were entrained in LD cycles in the same incubator, with lights-on at 06:30 h (ZT0) and lights-off at 18:30 (ZT12). Subsequently, during day 3, control flies were always in the same condition (12L:12D), but treated flies were exposed to discontinuous light stimulation (DLS) during scotophase. We used light interruption to treat flies (white light, 500 lux, and 0.008 mW/cm2) for one hour and intervals of one and half hours between treatments, with four repeats (treatment times: ZT13–14, ZT 15.5–16.5, ZT18–19 and ZT20.5–21.5) delivered daily for 4 days, during which their sleep was recorded. We choose this condition to make sure that CRY was fully degraded due to DLS but to maintain a rhythm in sleep. (TIF) [file pone.0105678.s001.tif]
